# Transversal Survey of Emergency Medicine Policy and Quality Metrics in Japan’s Regional Health Care Plans

**DOI:** 10.31662/jmaj.2022-0172

**Published:** 2023-05-22

**Authors:** Atsuyoshi Iida, Shinya Saito, Jun Hamada, Shunsuke Nakamura, Tsuyoshi Nojima, Hiromichi Naito, Takeshi Mikane

**Affiliations:** 1 Department of Emergency, Critical Care, and Disaster Medicine, Okayama University Graduate School of Medicine Dentistry and Pharmaceutical Sciences, Okayama, Japan; 2 Graduate School of Health Sciences, Okayama University Graduate School of Medicine Dentistry and Pharmaceutical Sciences, Okayama, Japan; 3 Department of Health and Welfare Services Management, Kawasaki University of Medical Welfare, Kurashiki, Japan; 4 Department of Emergency Medicine, Japanese Red Cross Okayama Hospital, Okayama, Japan

**Keywords:** emergency medicine, indicator, measure, quality assurance, quality improvement

## Abstract

**Introduction:**

It is essential to establish appropriate medical quality metrics and make improvements to safely and efficiently deliver optimum emergency medical services. The Ministry of Health, Labor and Welfare (MHLW) recommends prefectures to establish numerical quality metrics in their regional healthcare plans (RHCP). The 7th RHCP was issued by the MHLW in 2017 along with a notice of planning in covering the six-year period from 2018 to 2023. In this descriptive study, the emergency medicine policies in the 7th RHCP of each prefecture were analyzed from a quality improvement perspective.

**Method:**

The authors examined the chapters on emergency medicine in the RHCPs of 47 prefectural governments for the overall structure, cost-benefits, and connection to community-based integrated care systems. The type and number of clinical measures listed as numerical metrics and their classification methods were emphasized.

**Result:**

Regarding the overall plan structure, 40 prefectural governments began their description with an analysis of current surroundings. In total, 24 prefectural governments mentioned community-based integrated care systems but none mentioned cost-benefit analysis. Altogether, only 43 of 47 prefectural governments (91%) indicated numerical metrics. The maximum number of numerical targets for quality measures by prefecture was 19, the minimum was 0, and the median was 4 (IQR: 3-6.5); there were 220 metrics in total, with 82 structural, 96 process, and 42 outcome measures. Additionally, 13 prefectures (28%) classified quality measures according to the MHLW’s guidance, 6 (13%) used their own classification manner, while the others did not classify their measures.

**Conclusions:**

There were significant differences in emergency medicine policies and quality metrics among the prefectural governments. Further research is needed to develop and establish more comprehensive and appropriate metrics based on a common methodology to improve the quality of emergency medicine.

## Introduction

The demand for emergency medical services has been increasing in Japan. The number of emergency medical evacuation was approximately 5.98 million in 2019, being the highest since then ^(1)^. Emergency medical services ensure social security enjoyed by all citizens. Given the limited emergency medical resources, it is important to establish a centralized emergency medical service system through collaboration among local emergency medical institutions and related stakeholders to provide higher quality emergency medical care while meeting this demand.

Quality of healthcare implies “the degree to which health services for individuals and populations increase the likelihood of desired health outcomes and their consistency with current professional knowledge” ^(2)^. Emergency medicine is an area of healthcare wherein quality measures must be assessed for its improvement. Systematic collection and analysis of data must measure its quality, and the results must be presented explicitly in a plan with new numerical metrics. Studies have nationally examined quality measures, and these have demonstrated the improvements in quality in the United States and the United Kingdom ^(3), (4)^. Conversely, the Organization for Economic Co-operation and Development (OECD) reported that despite the high level medicine, efforts to assess and improve the quality of healthcare were fewer in Japan than in other countries in 2014 ^(5)^.

Experiences from countries with national quality policies and strategies―an organized effort by a country to support and plan for improved quality of care―emphasized the benefits of having a single, cohesive plan that offers guidance and direction on quality at all levels of the system. The local government is one of the institutions that can play an important role ^(6)^. To deliver quality medical services to all Japanese citizens, each prefecture must implement policies and strategies that are nationally consistent, transparent, and tailored to their particular environments.

Regional healthcare plans (RHCPs) refer to six-year plans by the Ministry of Health, Labor and Welfare (MHLW) that each prefecture is instructed to follow to establish an efficient medical system tailored to the particular conditions of each prefecture, so that patients can receive the same high-quality medical services throughout Japan. No independent study has focused on the emergency medicine chapters of every 7th RHCP initiated in 2018 to provide an overview and evaluate numerical quality metrics. Therefore, this study aimed to review the overall structure of chapters on emergency medicine in each RHCP and the listed numerical quality metrics to address the ideal quality assessment.

## Materials and Methods

Data were collected from the 7^th^ RHCPs of 47 Japanese prefectures by referring to the homepage of each prefectural government (Supplementary Table). In the RHCP formulation guides, the MHLW clarifies that it is important to strengthen the policy cycle mechanism for an efficient and high-quality healthcare delivery system in each region ^(7)^. To be more precise, each prefecture should include the following in its new RHCP: consider the health status of the population and patient outcomes, evaluate the current status of the healthcare delivery system, assess the current RHCP, identify issues based on each of the directions to be pursued and measures to resolve them, establish numerical metrics, and evaluate the progress of these goals ^(7)^. First, for the chapter on emergency medicine in each plan, the authors compared the overall paragraph structure, including the logical development and the positioning of quality measures. The presence of a reflection and analysis of the previous plan was also checked. The relevant chapter indicated whether it comprised a cost-benefit analysis and a connection to a community-based integrated care system, a comprehensive service for housing, medical care, nursing care, prevention, and lifestyle support by the local governments ^(8)^.

Additionally, information on the number of prefectures that have explicitly stated numerical metrics as goals; the number, type, and categorization of these metrics were collected. Only metrics explicitly stated as goals were included in this survey; measures listed as reference clinical measures were excluded.

The distribution of collected quality metrics in the classification of structure, process, and outcome (SPO) were also considered, which are the common classification of clinical measures proposed by Donabedian^(9), (10)^. The MHLW instructs prefectures to use Donabedian’s classification of quality in their RHCP formulation guides ^(7), (11)^; the Japan Council for Quality Healthcare uses this classification as one of its leading indicator definitions ^(12)^. When a prefectural government adopted an SPO classification for quality metrics, it was counted as such; otherwise, the authors classified them.

The self-reported classification by each RHCP was categorized as either medical stage classification or SPO classification given by MHLW ^(7)^, prefecture-unique classification, or no classification.

During data extraction, two authors, AI and SS, read all the documents, and after iterative discussions with JH, the number of quality metrics and the classification method for each prefecture were counted. Any conflicts in counting or classifying were reconciled through discussion until the authors reached a consensus.

## Result

The RHCP of each prefecture is available online only in Japanese. Regarding the organization of paragraphs in the chapter on the emergency medicine of each RHCP, 40 prefectural governments began their description with an “analysis of the current situation,” followed by “identification of issues,” “policies,” and “clinical metrics.” In five prefectures, the “current situation analysis” was preceded by a description of “what the prefecture should aim for.” Two prefectures evaluated the previous plan at the beginning.

Half of the prefectural governments―24 prefectural governments―mentioned a community-based integrated care system in emergency medicine chapter. Thus, these prefectural governments had a consistent overview of emergency medicine from the prehospital to the chronic stage. The remaining 23 prefectural governments described the prehospital to acute care stage without mentioning a community-based integrated care system. Cost-effectiveness analysis was not mentioned by all prefectural governments.

**Figure 1.**
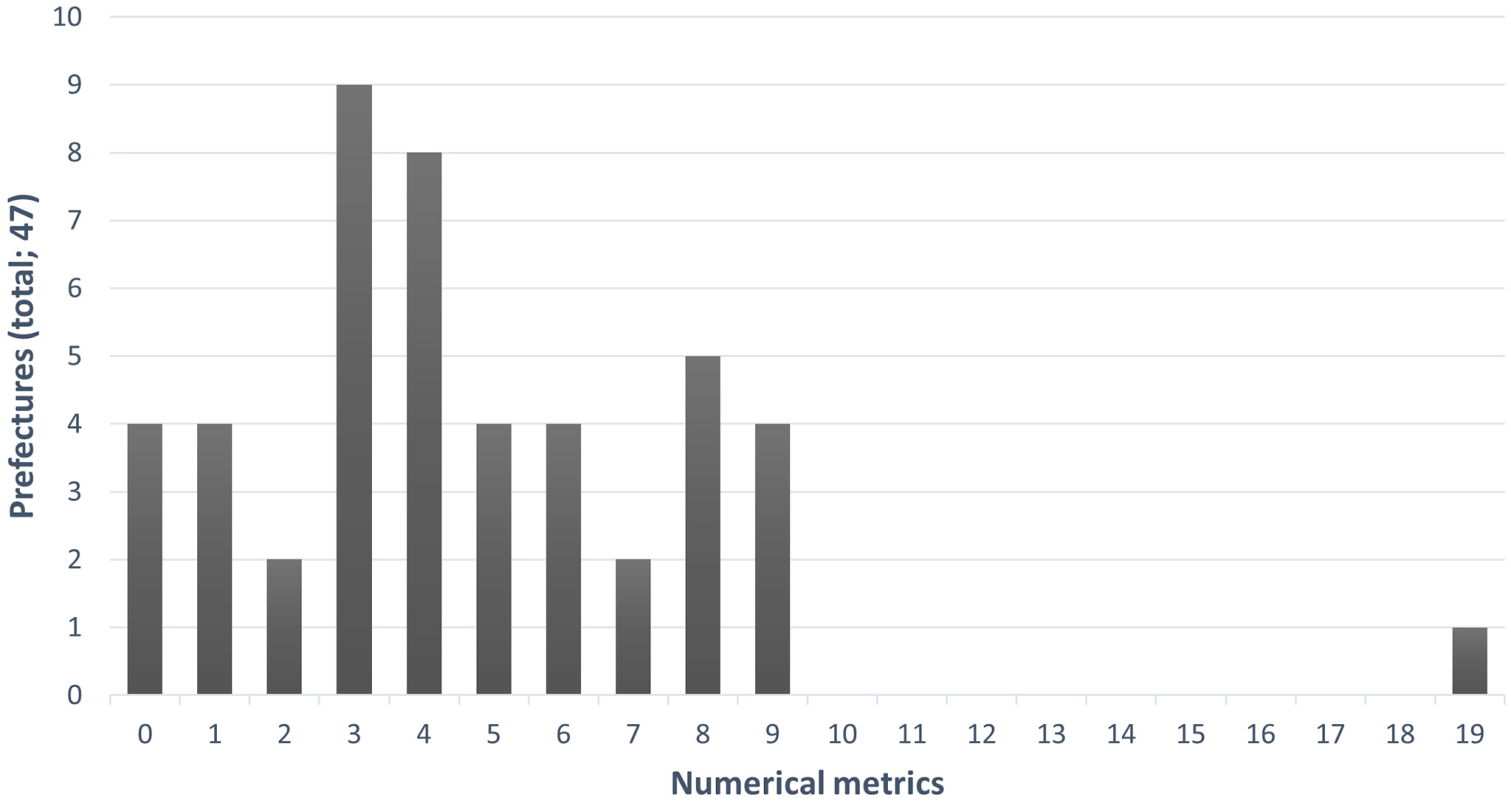
Distribution of the number of prefectural governments by the number of numerical metrics There were four prefectures with 0 numerical metrics and one with a maximum of 19. The median value was 4 (IQR: 3-6.5), and almost all prefectural governments had fewer than 10 measures.

Of the prefectures with target-setting clinical metrics, 43 (91%) numerically indicated their goals. Four prefectural governments (9%) did not indicate numerical targets. The number of clinical measures with stated goals by prefectural governments varied widely―from 0 in some prefectures to a maximum of 19 (Figure 1).

Among the prefectural governments with numerical targets, the number of numerical metrics was <10 for all prefectures except one. The median of metrics was 4 and IQR was 3-6.5. The total number of clinical quality metrics set by each prefectural government was 220. When classified by the SPO, there were 82 structural (37%), 96 process (44%), and 42 outcome (19%) measures. Proportionally, process metrics and outcome metrics were the highest and the lowest, respectively. The number of medical facilities providing emergency medical care, the duration of ambulance transport, and the percentage of patients who survived cardiac arrest after one month represented the corresponding SPO metrics.

**Figure 2.**
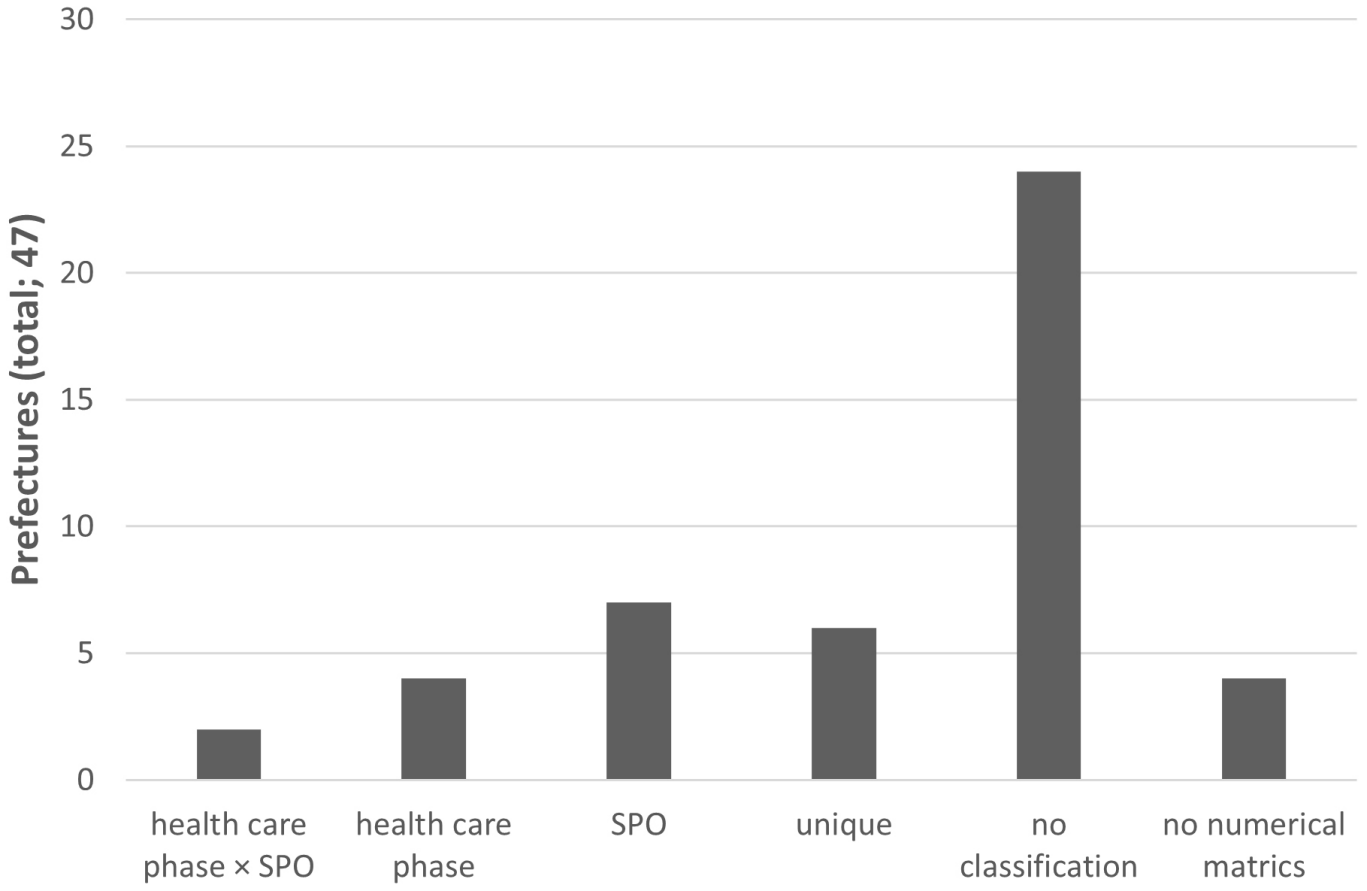
Distribution of the prefecture’s metric classification method Two prefectural governments used a contingency table of health care phase classification and SPO classification, seven used only SPO classification, and four used only health care phase classification. Six prefectural governments had their own unique classification. Twenty-four prefectural governments did not indicate any classification of their own numerical metrics.

Regarding each municipality’s categorized numerical metrics, two prefectural governments used a contingency table of healthcare phase classification and SPO classification, as shown in the example of the MHLW. Seven prefectural governments used only SPO classification and four used only the classification by healthcare phase. Six prefectural governments had their own unique classification. However, 24 prefectural governments, representing the majority, exempted any classification of their own numerical metrics (Figure 2).

These results are listed in the Table 1.

**Table 1.** The Number of Numerical Metrics, Classification, and Covering Community-Based Integrated Care System and Cost-Effectiveness Analysis by Prefecture.

Prefecture	Classification	Numerical metrics				Covered	
		Structure	Process	Outcome	Total	Community- based integrated care system	Cost-effectiveness analysis
**Hokkaido**	SPO	4	2	2	8	○	-
**Aomori**	Health care phase	1	3	0	4	○	-
**Iwate**	-	1	1	1	3	-	-
**Miyagi**	-	2	3	0	5	○	-
**Akita**	SPO	3	3	2	8	○	-
**Yamagata**	SPO	4	2	2	8	○	-
**Fukushima**	Health care phase	2	0	2	4	○	-
**Ibaraki**	-	1	5	2	8	○	-
**Tochigi**	-	0	5	0	5	○	-
**Gunma**	Health care phase	3	2	1	6	○	-
**Saitama**	-	0	3	0	3	-	-
**Chiba**	SPO	4	3	2	9	-	-
**Tokyo**	Unique	0	6	0	6	○	-
**Kanagawa**	-	0	3	0	3	○	-
**Niigata**	-	0	2	0	2	○	-
**Toyama**	-	1	3	1	5	-	-
**Ishikawa**	-	1	1	1	3	-	-
**Fukui**	Health care phase × SPO	0	3	1	4	-	-
**Yamanashi**	-	0	0	0	0	-	-
**Nagano**	Health care phase × SPO	4	1	1	6	○	-
**Gifu**	SPO	2	5	2	9	○	-
**Shizuoka**	-	0	1	2	3	-	-
**Aichi**	-	1	0	0	1	○	-
**Mie**	-	1	3	0	4	○	-
**Shiga**	-	3	1	0	4	-	-
**Kyoto**	-	3	3	2	8	-	-
**Osaka**	Unique	3	2	1	6	-	-
**Hyogo**	-	1	0	0	1	-	-
**Nara**	-	0	1	0	1	○	-
**Wakayama**	Unique	2	5	0	7	○	-
**Tottori**	-	0	1	0	1	-	-
**Shimane**	-	3	0	0	3	-	-
**Okayama**	-	0	0	0	0	-	-
**Hiroshima**	SPO	2	3	4	9	○	-
**Yamaguchi**	-	1	1	0	2	○	-
**Tokushima**	-	4	3	0	7	○	-
**Kagawa**	-	0	0	0	0	-	-
**Ehime**	-	0	0	3	3	○	-
**Kochi**	SPO	1	4	0	5	-	-
**Fukuoka**	-	0	1	2	3	○	-
**Saga**	Unique	2	5	2	9	-	-
**Nagasaki**	SPO	1	2	1	4	-	-
**Kumamoto**	-	0	1	2	4	-	-
**Oita**	-	3	1	0	4	-	-
**Miyazaki**	-	0	3	0	3	-	-
**Kagoshima**	-	0	0	0	0	-	-
**Okinawa**	Unique	6	11	1	18	○	-

## Discussion

In the Unites States and the United Kingdom, quality improvement activities are carried out using a centralized approach ^(3), (4)^. However, in Japan, the efforts are limited to hospital groups, and a nationwide centralized approach is still in the nascent stages ^(12)^. Additionally, the national quality improvement approach focuses on medical facility units individually rather than regionally or nationally. There has been no quality improvement analysis of RHCPs from a nationwide perspective. This paper presents the first independent evaluation of quality metrics for RHCP emergency medicine chapters.

RHCP must analyze healthcare-related data in the prefectures based on quality measures, identify problems in the healthcare provision system, and present a reasonable and concrete system of measures.

It should also evaluate the results and improve the content through a quality control cycle ^(13)^. This study clarified that there is considerable variation in the method and description of the quality of emergency medicine in the RHCPs formulated by each prefecture in Japan, which highlights that there is no unified strategy for quality assurance and improvement in emergency medicine.

According to the MHLW’s instructions in the RHCP formulation guides, each prefecture should clearly state its policies to address the issues, set numerical metrics, and classify these numerical metrics as per Donabedian’s SPO classification ^(7), (11)^. Therefore, the authors investigated the application of Donabedian’s SPO classification; in case Donabedian’s SPO classification was not used, which classification method was used. Furthermore, the MHLW has requested each prefecture to improve the RHCP to create a medical system that offers high-quality, patient-centered, and efficient care ^(7)^. According to Hansen et al., “there is an urgent need to improve the evidence based medicine to determine which quality indicators have the potential to economically improve clinical outcomes between staff and patient experience and to develop indicators that will guide practice improvement” ^(14)^. Given these rationales and Japan’s fiscal environment, incorporating and outlining a cost-benefit analysis in the RHCP would be beneficial to residents.

Regarding the overall structure of the analyzed chapters, 85% (40/47) of the prefectural governments began their descriptions with an analysis of the ongoing situation, following the MHLW guidelines ^(7)^. Consistency in the recognition of the current situation, objectives, and means is important, and the plan for the current term should be formulated based on the evaluation and reflection of the previous term’s plan, ensuring continuity in administrative measures. The same guidelines also mention the importance of the Plan-Do-Check-Act (PDCA) cycle ^(7)^. Thus, the two prefectural governments that described the “evaluation of the previous plan” independently are worthy of special mention. These two prefectures have based their RHCPs on MHLW guidelines and exercised ingenuity for better elucidation. Furthermore, five prefectural governments adopted the structure of presenting a grand design before delving into individual analysis.

Only half of the prefectural governments mentioned a community-based integrated care system in their chapter on emergency medicine. Hansen et al. recommend that the series of measures for the emergency medical system should cover the entire patient pathway, from prehospital to post-discharge, beyond the acute care hospital ^(14)^. The MHLW also urges each prefecture to ensure that their emergency medical care system contributes to a community-based integrated care system ^(11)^. Improving the system is necessary to cope with changes in the socioeconomic structure, along with timely and practical collaborations with community-based integrated care systems in emergency medicine policies. Moreover, to implement the PDCA cycle of the RHCP and contribute to the health of local residents, it is essential to have a set of concrete measures to resolve issues and bring the budget necessary for the implementation of projects ^(15)^. As efforts to improve the delivery of medical care continue worldwide, medical delivery systems need to be properly scaled and adapted to local needs and socioeconomic conditions for maximum effectiveness ^(16)^. Changes in the socioeconomic structure mean that medical and financial resources are also finite, leading to prioritization and efficiency considerations for emergency medical policy goals. Verification of the cost-effectiveness analysis is necessary for efficient measures; although, a cost-effectiveness analysis was not mentioned by any municipality.

The number of clinical quality metrics that stated goals was widely distributed. For facilitating the PDCA cycle, along with appropriate and objective assessment, establishing numerical metrics is essential. However, four prefectural governments (9% of all prefectures) did not indicate numerical metrics. Hence, there is no objective criterion to visualize whether the measures taken to improve emergency medical services have been effective when the next plan is formulated. The aforementioned four prefectural governments should establish numerical metrics when the next RHCP is formulated. However, if there are too many quality measures, the priorities and goals of the measures may not be determined, and the plan may be imbalanced ^(15)^. Prefectural governments that list various issues in the RHCP but have only a few numerical metrics must prioritize regional surroundings or cost-benefit analysis. The MHLW listed 22 examples of measures, 5 of which are designated as priorities ^(17)^. While none of the prefectures have implemented all 22 of the measures outlined in the MHLW, 40 (85%) have adopted at least one of these measures. Regarding the five priority measures, while none of the priority measures was adopted by all prefectures, 38 (81%) adopted at least one. Although it is generally difficult to determine a reasonable number of numerical metrics because of the actual circumstances and resources of each region, a range of 5-10 appears realistic.

Of the 43 prefectural governments that clearly stated numerical metrics, only 44% (19 governments) used some type of measures classification method. One of the leading measures used extensively is the classification proposed by Donabedian, which relates to the SPO of healthcare ^(9), (10)^. “Structure” indicates the attributes of the environment wherein healthcare is provided. “Process” refers to what actually takes place in the provision of healthcare, including activities such as diagnoses and recommending/implementing treatments. “Outcome” is the effect of the healthcare implemented on the health status of the patient or population. The MHLW illustrates a contingency table between the aforementioned classification and classification by medical phase: prehospital care, primary and intensive treatment in the hospital, and post-acute phase care ^(17)^. Two prefectural governments have adopted this. Additionally, there are six RHCPs whose numerical metrics are categorized according to each municipality’s own unique classification. This is interesting because it indicates the thoughts and tactics of prefectural government officials. Each municipality’s classification of its own numerical metrics should reflect the thought patterns of officials when establishing policies. There are few caveats when using Donabedian’s SPO classification. Only process measures that have been proven to lead to better outcomes are valid. Similarly, structural measures can be used for quality assurance but only if shown to increase the likelihood of good outcomes or related processes shown to produce good outcomes ^(18)^. Mostly, multiple factors contribute to patient survival and health outcomes. Therefore, risk adjustment is essentially important when comparing outcome measures across hospitals and regions ^(19), (20), (21), (22)^. For example, the one month survival rates of out-of-hospital cardiac arrest (OHCA), adopted by 19 prefectures as a numerical target, should be adjusted for regional aging proportion. Additionally, if advance directives are widespread, patients’ families may request resuscitation to be halted at an early stage. These factors are not revealed by simply comparing the survival rates of OHCA. Outcome measures have some limitations but can be useful for screening areas that require detailed assessments. A retrospective evaluation of the process that leads to undesirable outcomes is necessary to improve quality. Exploration of process improvements may also lead to the evaluation of causal structural features ^(23)^. Indeed, process measures were the most common while outcome measures were the least common among the numerical metrics established by prefectural governments; this demonstrates that the authorities are not fixated on the outcome but on structural and process measures.

This study revealed that there are significant differences in emergency medicine policies and quality metrics among the prefectural governments. Valuable metrics should be based on the best available evidence with properties of repeatability and consistency. They are also related with the issue of technical feasibility and whether stakeholders are convinced by the measurement methods and consider the results in determining the quality of healthcare ^(23)^. Sackett et al. described this as “the integration of best research evidence with clinical expertise and patient values” ^(24)^. This quality measurement and improvement effort is comes with cost ^(25)^. These findings indicate that healthcare policies and measurements also need to be viewed from the perspectives of efficiency and equity.

The quality of health systems includes the healthcare service provision settings as a significant aspect ^(12)^. The RHCP, a healthcare plan developed by each regional government, is the subject of this paper, which is situated in a policy context. This paper cites research on quality in healthcare service provision settings and research on quality in health systems, both of which can be regarded as healthcare quality indicators. Indeed, the measures established in the RHCPs combine the perspectives of quality in the healthcare service delivery setting and quality in the healthcare system. Finally, it may be important for RHCP to organize and distinguish between the two to develop more sophisticated and systematized quality indicators.

Furthermore, although MHLW mentions Donabedian’s SPO classification and PDCA cycle in its guidelines for RHCP development ^(10)^, other perspectives in the development of quality measures should also be presented. For instance, the Agency for Healthcare Research and Quality in the United States defines the “Six Domains of Health Care Quality” as ^(26)^ “safe,” “effective,” “patient-centered,” “timely,” “efficient,” and “equitable.” These cover the basic principles in providing high-quality medical services common to all fields of practice ^(27)^. Hansen et al. proposed that defining SPO quality measures in each of the six domains is an appropriate and reasonable framework for improving quality in the International Federation for Emergency Medicine ^(14)^. The actual measures based on these domains should depend on local surroundings, data availability, and the healthcare system. According to the Japan Council for Quality Health Care’s 2022 guidance ^(12)^, which adopts the OECD Health Care Quality Indicator framework, the three primary characteristics of high-quality healthcare are effectiveness, safety, and responsiveness. Efficiency, access, and equity were also highlighted as characteristics of the healthcare system. It also encourages the establishment of measures for each of them along with medical stage (health care needs) classifications such as primary prevention, acute care, chronic care, and end-of-life care. This view of healthcare quality aligns with WHO’s 2018 guidance ^(5)^ and is the current global standard. The MHLW should direct prefectures to set metrics in their upcoming RHCPs based on this framework and to disclose whether the PDCA cycle is functioning properly once those metrics have been set.

Measurements in RHCP are a means of assessing the current status of the medical care delivery system in a prefecture, and the key to effective functioning is the integrated operation of metrics and policies ^(13)^. Physicians should focus on the current state of quality measurement in emergency medicine, particularly the process of determining quality metrics that affect individual healthcare providers.

In conclusion, the chapter on emergency medicine in the RHCP of each prefecture showed significant differences among prefectural governments in terms of composition, the number of numerical metrics for quality improvement, and classification perspective. Although each municipality has its own unique circumstances, common methodologies for quality improvement are necessary to develop quality measures and set goals to assure quality improvement. Further research with a health policy perspective that contributes to the establishment of more comprehensive and appropriate metrics is required to improve the quality of emergency medical services.

## Article Information

### Conflicts of Interest

None

### Acknowledgement

We would like to thank Editage (www.editage.com) for English language editing.

### Author Contributions

Survey design: AI, SS, JH; data collection and analysis: AI; manuscript preparation: AI, SS, SN, TN, HN; survey supervision: TM

### Approval by Institutional Review Board (IRB)

Not applicable

### Data Availability Statement

The datasets used and/or analyzed during the current survey are available from the corresponding author on reasonable request.

## Supplement

Supplementary Table
