# Supplementary material for: Transversal Survey of Emergency Medicine Policy and Quality Metrics in Japan’s Regional Health Care Plans
Source: JMA J. 2023 May 22;6(3):284–91. doi: 10.31662/jmaj.2022-0172 (PMC10407299; doi:10.31662/jmaj.2022-0172)
Supplement: Supplementary Table [file 2433-3298-6-3-0284-s001.pdf]

Supplementary table: Web address containing regional health care plans for each prefecture

Prefecture	Web address
**Hokkaido**	https://www.pref.hokkaido.lg.jp/hf/cis/iryokeikaku/aratanairyoukeikaku.html
**Aomori**	https://www.pref.aomori.lg.jp/soshiki/kenko/iryo/iryo_plan_h30.html
**Iwate**	https://www.pref.iwate.jp/kurashikankyou/iryou/seido/keikaku/1002862.html
**Miyagi**	https://www.pref.miyagi.jp/soshiki/iryou/rmpindex.html
**Akita**	https://www.pref.akita.lg.jp/pages/archive/3120
**Yamagata**	https://www.pref.yamagata.jp/090001/kenfuku/iryo/keikaku/hokeniryou-plan-62.html
**Fukushima**	https://www.pref.fukushima.lg.jp/sec/21045c/iryou-keikaku7.html
**Ibaraki**	https://www.pref.ibaraki.jp/hokenfukushi/iryo/keikaku/koso/health-med-plan/index.html
**Tochigi**	https://www.pref.tochigi.lg.jp/e02/pref/keikaku/bumon/hokeniryou7.html
**Gunma**	https://www.pref.gunma.jp/02/d10g_00039.html
**Saitama**	https://www.pref.saitama.lg.jp/a0701/iryou-keikaku/keikakunaiyou.html
**Chiba**	https://www.pref.chiba.lg.jp/kenfuku/keikaku/kenkoufukushi/30hokeniryou.html
**Tokyo**	https://www.fukushihoken.metro.tokyo.lg.jp/iryo/iryo_hoken/hoken_keikaku.html

**Kanagawa**	https://www.pref.kanagawa.jp/docs/t3u/cnt/f742/dainanaji.html
**Niigata**	https://www.pref.niigata.lg.jp/sec/chiikiiryo/1356890019056.html
**Toyama**	https://www.pref.toyama.jp/1204/kurashi/kenkou/iryou/kj00006481/index.html
**Ishikawa**	https://www.pref.ishikawa.lg.jp/iryou/support/iryoukeikaku/iryoukeikaku.html
**Fukui**	https://www.pref.fukui.lg.jp/doc/iryou/iryoujouhou/7ji-iryoukeikaku.html
**Yamanashi**	https://www.pref.yamanashi.jp/imuka/42_002.html
**Nagano**	https://www.pref.nagano.lg.jp/kenko-fukushi/kenko/iryo/shisaku/2ndsogokeikaku2.html
**Gifu**	https://www.pref.gifu.lg.jp/page/124969.html
**Shizuoka**	http://www.pref.shizuoka.jp/kousei/ko-410/hi-keikaku.html
**Aichi**	https://www.pref.aichi.jp/soshiki/iryo-keikaku/iryokeikaku.html
**Mie**	https://www.pref.mie.lg.jp/IRYOS/HP/24199023348_00001.htm
**Shiga**	https://www.pref.shiga.lg.jp/ippan/kenkouiryouhukushi/iryo/300043.html
**Kyoto**	https://www.pref.kyoto.jp/hofukuki/
**Osaka**	https://www.pref.osaka.lg.jp/iryo/keikaku/7osakahuiryokeikaku.html
**Hyogo**	https://web.pref.hyogo.lg.jp/kf15/keikaku2018.html

**Nara**	https://www.pref.nara.jp/2740.htm
**Wakayama**	https://www.pref.wakayama.lg.jp/prefg/050100/iryokeikaku/keikaku.html
**Tottori**	https://www.pref.tottori.lg.jp/274573.htm
**Shimane**	https://www.pref.shimane.lg.jp/medical/kenko/iryo/shimaneno_iryo/hokenniryoukeikaku/
**Okayama**	https://www.pref.okayama.jp/page/710082.html
**Hiroshima**	https://www.pref.hiroshima.lg.jp/soshiki/64/hokeniryoukeikaku-7.html
**Yamaguchi**	https://www.pref.yamaguchi.lg.jp/soshiki/45/14255.html
**Tokushima**	https://www.pref.tokushima.lg.jp/ippannokata/kenko/iryo/5014521
**Kagawa**	https://www.pref.kagawa.lg.jp/imu/kenmin/wh5mp3200227162055.html
**Ehime**	https://www.pref.ehime.jp/h20150/keikaku/keikaku/dai7jiiryokaikaku.html
**Kochi**	https://www.pref.kochi.lg.jp/soshiki/131301/2018032800404.html
**Fukuoka**	https://www.pref.fukuoka.lg.jp/contents/hoken-iryo-keikaku-2018.html
**Saga**	https://www.pref.saga.lg.jp/kiji00385414/index.html
**Nagasaki**	https://www.pref.nagasaki.jp/bunrui/hukushi-hoken/iryo/keikaku-iryo/iryoukeikaku/
**Kumamoto**	https://www.pref.kumamoto.jp/soshiki/27/4723.html

**Oita**	https://www.pref.oita.jp/soshiki/12620/iryoukeikaku.html
**Miyazaki**	https://www.pref.miyazaki.lg.jp/iryoyakumu/kense/kekaku/20220329143037.html
**Kagoshima**	https://www.pref.kagoshima.jp/ae01/kenko-fukushi/kenko-iryo/iryokeikaku/keikaku30-3.html
**Okinawa**	https://www.pref.okinawa.jp/site/hoken/iryoseisaku/kikaku/iryoukeikaku.html
